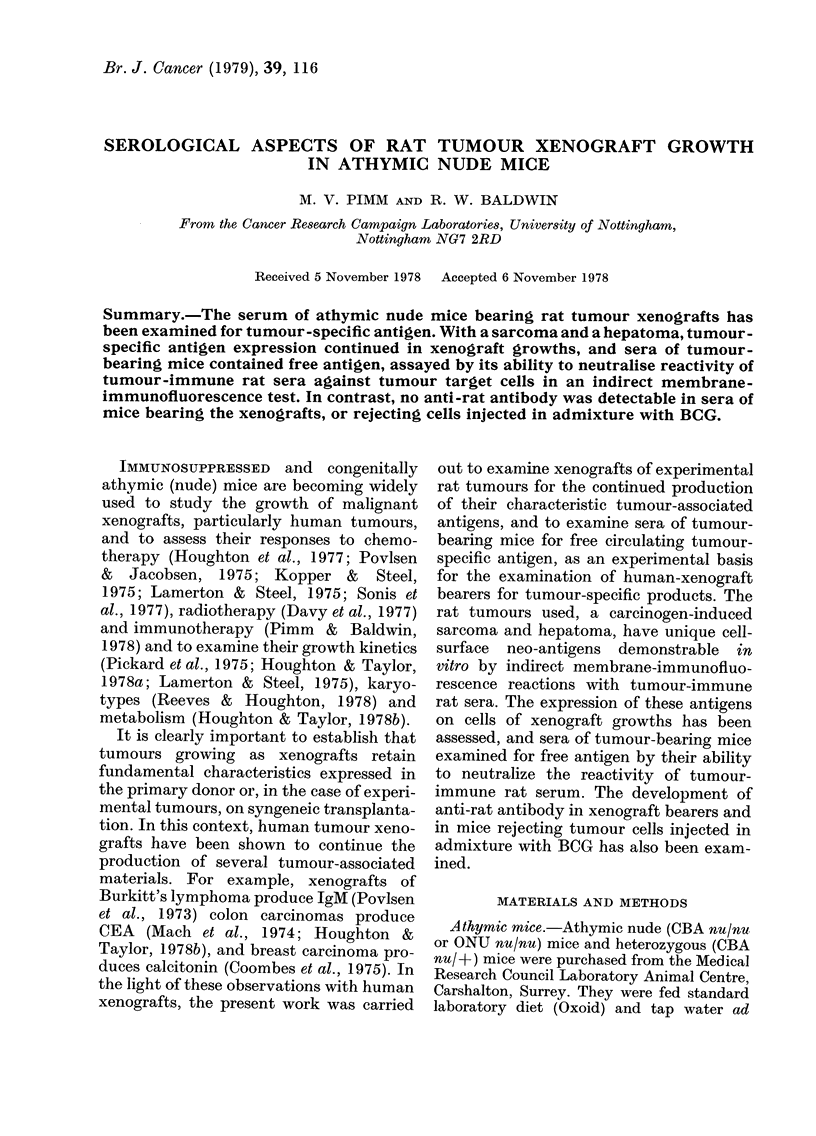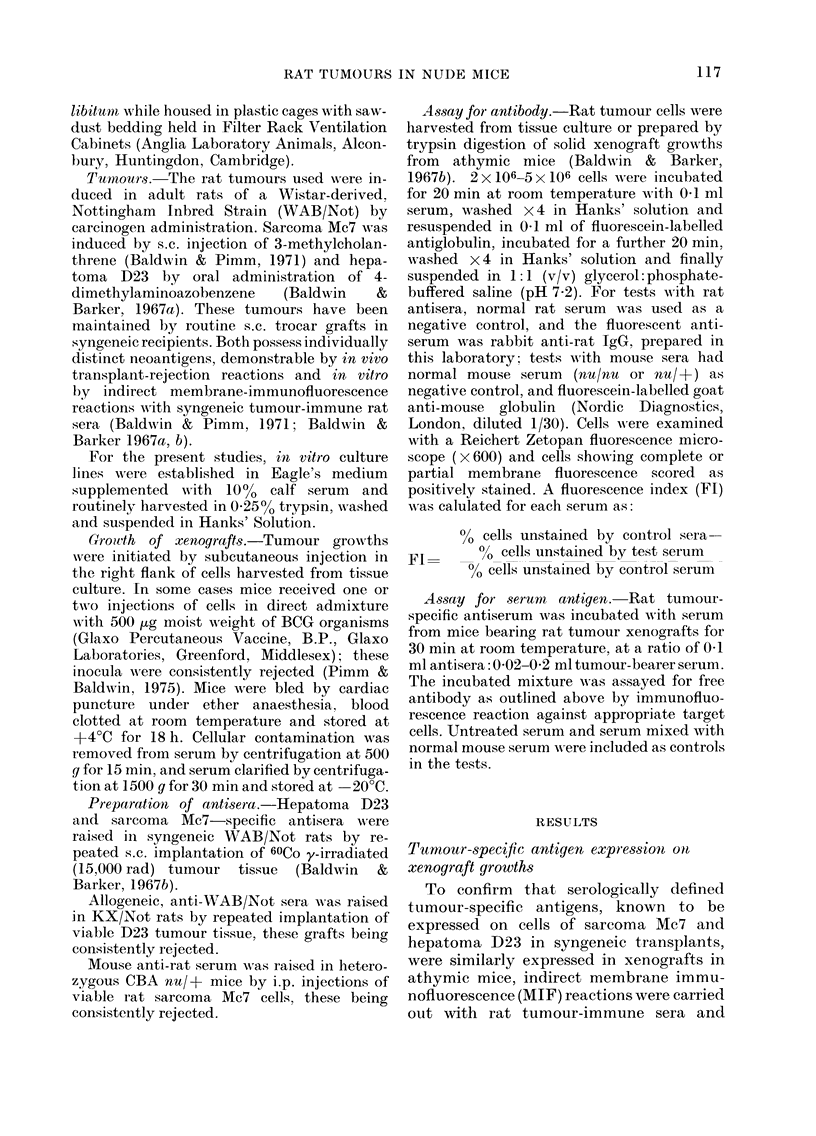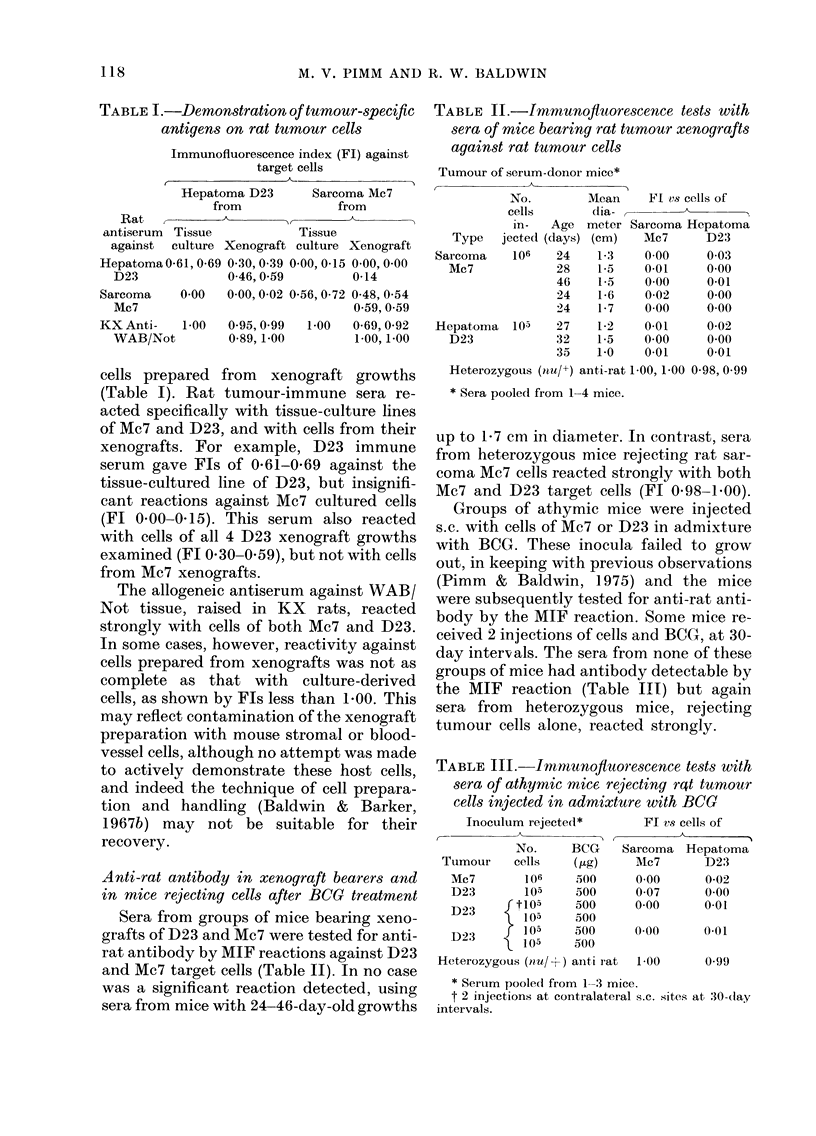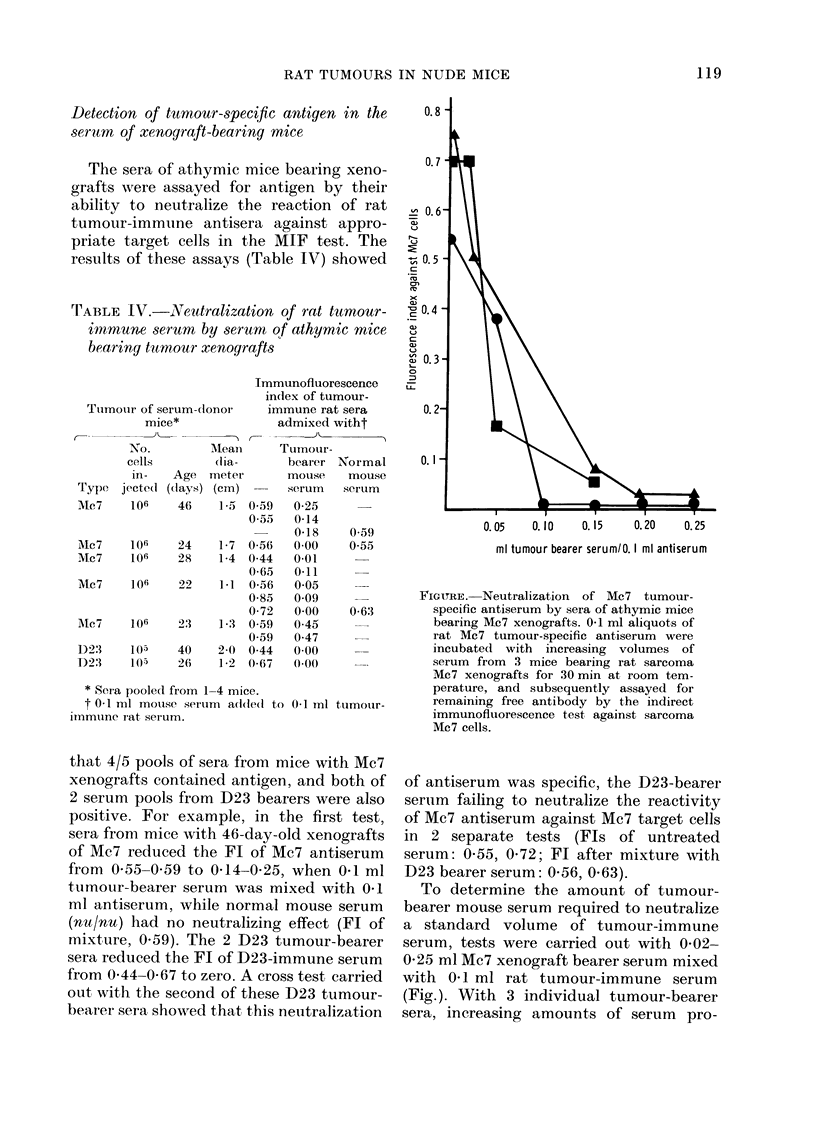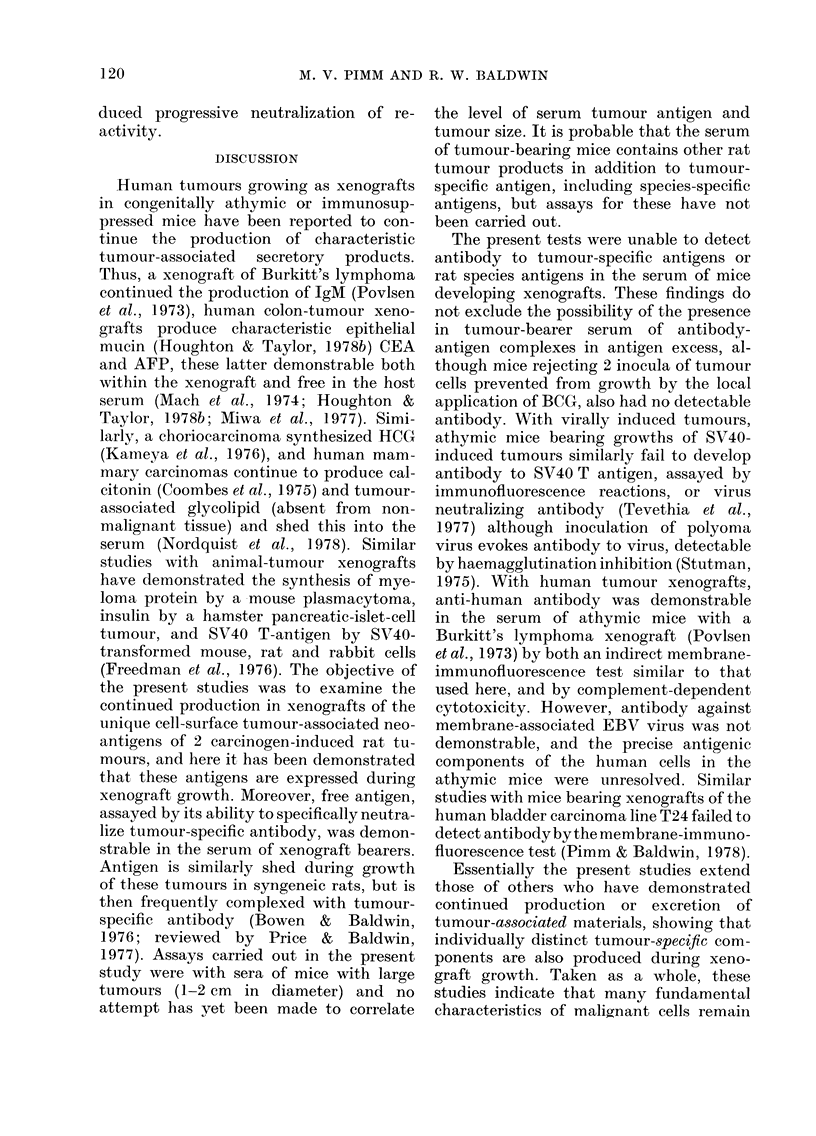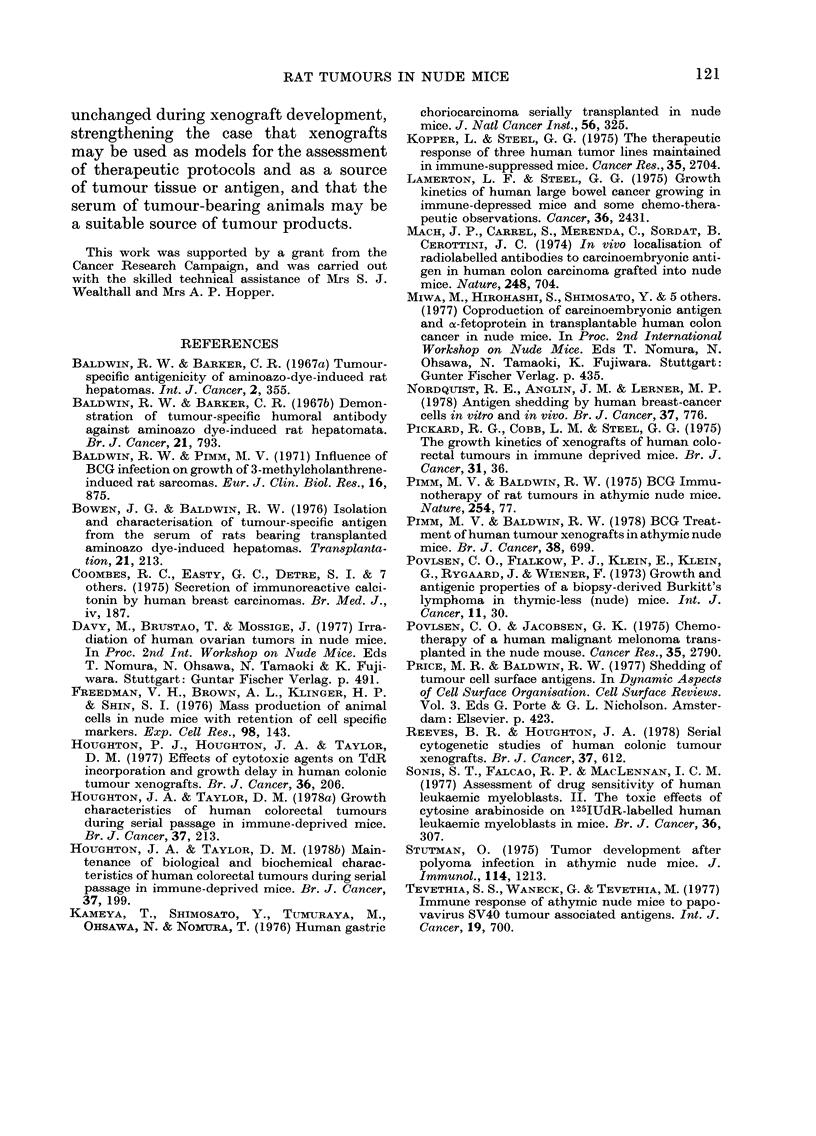# Serological aspects of rat tumour xenograft growth in athymic nude mice.

**DOI:** 10.1038/bjc.1979.21

**Published:** 1979-02

**Authors:** M. V. Pimm, R. W. Baldwin

## Abstract

The serum of athymic nude mice bearing rat tumour xenografts has been examined for tumour-specific antigen. With a sarcoma and a hepatoma, tumour-specific antigen expression continued in xenograft growths, and sera of tumour-bearing mice contained free antigen, assayed by its ability to neutralise reactivity of tumour-immune rat sera against tumour target cells in an indirect membrane-immunofluorescence test. In contrast, no anti-rat antibody was detectable in sera of mice bearing the xenografts, or rejecting cells injected in admixture with BCG.


*Br. J. Cancer* (1979), **39**, 116

### SEROLOGICAL ASPECTS OF RAT TUMOUR XENOGRAFT GROWTH IN ATHYMIC NUDE MICE

M. V. PIMM AND R. W. BALDWIN

*From the Cancer Research Campaign Laboratories, University of Nottingham, Nottingham NG7 2RD*

Received 5 November 1978 Accepted 6 November 1978

**Summary.—The serum of athymic nude mice bearing rat tumour xenografts has been examined for tumour-specific antigen. With a sarcoma and a hepatoma, tumour-specific antigen expression continued in xenograft growths, and sera of tumour-bearing mice contained free antigen, assayed by its ability to neutralise reactivity of tumour-immune rat sera against tumour target cells in an indirect membrane-immunofluorescence test. In contrast, no anti-rat antibody was detectable in sera of mice bearing the xenografts, or rejecting cells injected in admixture with BCG.**

IMMUNOSUPPRESSED and congenitally athymic (nude) mice are becoming widely used to study the growth of malignant xenografts, particularly human tumours, and to assess their responses to chemotherapy (Houghton *et al.*, 1977; Povlsen & Jacobsen, 1975; Kopper & Steel, 1975; Lamerton & Steel, 1975; Sonis *et al.*, 1977), radiotherapy (Davy *et al.*, 1977) and immunotherapy (Pimm & Baldwin, 1978) and to examine their growth kinetics (Pickard *et al.*, 1975; Houghton & Taylor, 1978*a*; Lamerton & Steel, 1975), karyotypes (Reeves & Houghton, 1978) and metabolism (Houghton & Taylor, 1978*b*).

It is clearly important to establish that tumours growing as xenografts retain fundamental characteristics expressed in the primary donor or, in the case of experimental tumours, on syngeneic transplantation. In this context, human tumour xenografts have been shown to continue the production of several tumour-associated materials. For example, xenografts of Burkitt's lymphoma produce IgM (Povlsen *et al.*, 1973) colon carcinomas produce CEA (Mach *et al.*, 1974; Houghton & Taylor, 1978*b*), and breast carcinoma produces calcitonin (Coombes *et al.*, 1975). In the light of these observations with human xenografts, the present work was carried out to examine xenografts of experimental rat tumours for the continued production of their characteristic tumour-associated antigens, and to examine sera of tumour-bearing mice for free circulating tumour-specific antigen, as an experimental basis for the examination of human-xenograft bearers for tumour-specific products. The rat tumours used, a carcinogen-induced sarcoma and hepatoma, have unique cell-surface neo-antigens demonstrable *in vitro* by indirect membrane-immunofluorescence reactions with tumour-immune rat sera. The expression of these antigens on cells of xenograft growths has been assessed, and sera of tumour-bearing mice examined for free antigen by their ability to neutralize the reactivity of tumour-immune rat serum. The development of anti-rat antibody in xenograft bearers and in mice rejecting tumour cells injected in admixture with BCG has also been examined.

### MATERIALS AND METHODS

*Athymic mice.*—Athymic nude (CBA *nu/nu* or ONU *nu/nu*) mice and heterozygous (CBA *nu/+*) mice were purchased from the Medical Research Council Laboratory Animal Centre, Carshalton, Surrey. They were fed standard laboratory diet (Oxoid) and tap water *ad*

*libitum* while housed in plastic cages with sawdust bedding held in Filter Rack Ventilation Cabinets (Anglia Laboratory Animals, Alconbury, Huntingdon, Cambridge).

*Tumours.*—The rat tumours used were induced in adult rats of a Wistar-derived, Nottingham Inbred Strain (WAB/Not) by carcinogen administration. Sarcoma Mc7 was induced by s.c. injection of 3-methylcholanthrene (Baldwin & Pimm, 1971) and hepatoma D23 by oral administration of 4-dimethylaminoazobenzene (Baldwin & Barker, 1967*a*). These tumours have been maintained by routine s.c. trocar grafts in syngeneic recipients. Both possess individually distinct neoantigens, demonstrable by *in vivo* transplant-rejection reactions and *in vitro* by indirect membrane-immunofluorescence reactions with syngeneic tumour-immune rat sera (Baldwin & Pimm, 1971; Baldwin & Barker 1967*a*, *b*).

For the present studies, *in vitro* culture lines were established in Eagle's medium supplemented with 10% calf serum and routinely harvested in 0·25% trypsin, washed and suspended in Hanks' Solution.

*Growth of xenografts.*—Tumour growths were initiated by subcutaneous injection in the right flank of cells harvested from tissue culture. In some cases mice received one or two injections of cells in direct admixture with 500 μg moist weight of BCG organisms (Glaxo Percutaneous Vaccine, B.P., Glaxo Laboratories, Greenford, Middlesex); these inocula were consistently rejected (Pimm & Baldwin, 1975). Mice were bled by cardiac puncture under ether anaesthesia, blood clotted at room temperature and stored at +4°C for 18 h. Cellular contamination was removed from serum by centrifugation at 500 *g* for 15 min, and serum clarified by centrifugation at 1500 *g* for 30 min and stored at −20°C.

*Preparation of antisera.*—Hepatoma D23 and sarcoma Mc7—specific antisera were raised in syngeneic WAB/Not rats by repeated s.c. implantation of $^{60}Co$ γ-irradiated (15,000 rad) tumour tissue (Baldwin & Barker, 1967*b*).

Allogeneic, anti-WAB/Not sera was raised in KX/Not rats by repeated implantation of viable D23 tumour tissue, these grafts being consistently rejected.

Mouse anti-rat serum was raised in heterozygous CBA *nu*/+ mice by i.p. injections of viable rat sarcoma Mc7 cells, these being consistently rejected.

*Assay for antibody.*—Rat tumour cells were harvested from tissue culture or prepared by trypsin digestion of solid xenograft growths from athymic mice (Baldwin & Barker, 1967*b*). $2 \times 10^6$–$5 \times 10^6$ cells were incubated for 20 min at room temperature with 0·1 ml serum, washed ×4 in Hanks' solution and resuspended in 0·1 ml of fluorescein-labelled antiglobulin, incubated for a further 20 min, washed ×4 in Hanks' solution and finally suspended in 1:1 (v/v) glycerol:phosphate-buffered saline (pH 7·2). For tests with rat antisera, normal rat serum was used as a negative control, and the fluorescent antiserum was rabbit anti-rat IgG, prepared in this laboratory; tests with mouse sera had normal mouse serum (*nu*/*nu* or *nu*/+) as negative control, and fluorescein-labelled goat anti-mouse globulin (Nordic Diagnostics, London, diluted 1/30). Cells were examined with a Reichert Zetopan fluorescence microscope (×600) and cells showing complete or partial membrane fluorescence scored as positively stained. A fluorescence index (FI) was calulated for each serum as:

$$FI = \frac{\text{\% cells unstained by control sera} - \text{\% cells unstained by test serum}}{\text{\% cells unstained by control serum}}$$

*Assay for serum antigen.*—Rat tumour-specific antiserum was incubated with serum from mice bearing rat tumour xenografts for 30 min at room temperature, at a ratio of 0·1 ml antisera:0·02–0·2 ml tumour-bearer serum. The incubated mixture was assayed for free antibody as outlined above by immunofluorescence reaction against appropriate target cells. Untreated serum and serum mixed with normal mouse serum were included as controls in the tests.

### RESULTS

### *Tumour-specific antigen expression on xenograft growths*

To confirm that serologically defined tumour-specific antigens, known to be expressed on cells of sarcoma Mc7 and hepatoma D23 in syngeneic transplants, were similarly expressed in xenografts in athymic mice, indirect membrane immunofluorescence (MIF) reactions were carried out with rat tumour-immune sera and

TABLE I.—*Demonstration of tumour-specific antigens on rat tumour cells*

| Rat antiserum against | Immunofluorescence index (FI) against target cells: Hepatoma D23 from Tissue culture | Xenograft | Sarcoma Mc7 from Tissue culture | Xenograft |
|---|---|---|---|---|
| Hepatoma D23 | 0·61, 0·69 | 0·30, 0·39 0·46, 0·59 | 0·00, 0·15 | 0·00, 0·00 0·14 |
| Sarcoma Mc7 | 0·00 | 0·00, 0·02 | 0·56, 0·72 | 0·48, 0·54 0·59, 0·59 |
| KX Anti-WAB/Not | 1·00 | 0·95, 0·99 0·89, 1·00 | 1·00 | 0·69, 0·92 1·00, 1·00 |

cells prepared from xenograft growths (Table I). Rat tumour-immune sera reacted specifically with tissue-culture lines of Mc7 and D23, and with cells from their xenografts. For example, D23 immune serum gave FIs of 0·61–0·69 against the tissue-cultured line of D23, but insignificant reactions against Mc7 cultured cells (FI 0·00–0·15). This serum also reacted with cells of all 4 D23 xenograft growths examined (FI 0·30–0·59), but not with cells from Mc7 xenografts.

The allogeneic antiserum against WAB/Not tissue, raised in KX rats, reacted strongly with cells of both Mc7 and D23. In some cases, however, reactivity against cells prepared from xenografts was not as complete as that with culture-derived cells, as shown by FIs less than 1·00. This may reflect contamination of the xenograft preparation with mouse stromal or blood-vessel cells, although no attempt was made to actively demonstrate these host cells, and indeed the technique of cell preparation and handling (Baldwin & Barker, 1967*b*) may not be suitable for their recovery.

*Anti-rat antibody in xenograft bearers and in mice rejecting cells after BCG treatment*

Sera from groups of mice bearing xenografts of D23 and Mc7 were tested for anti-rat antibody by MIF reactions against D23 and Mc7 target cells (Table II). In no case was a significant reaction detected, using sera from mice with 24–46-day-old growths up to 1·7 cm in diameter. In contrast, sera from heterozygous mice rejecting rat sarcoma Mc7 cells reacted strongly with both Mc7 and D23 target cells (FI 0·98–1·00).

Groups of athymic mice were injected s.c. with cells of Mc7 or D23 in admixture with BCG. These inocula failed to grow out, in keeping with previous observations (Pimm & Baldwin, 1975) and the mice were subsequently tested for anti-rat antibody by the MIF reaction. Some mice received 2 injections of cells and BCG, at 30-day intervals. The sera from none of these groups of mice had antibody detectable by the MIF reaction (Table III) but again sera from heterozygous mice, rejecting tumour cells alone, reacted strongly.

TABLE II.—*Immunofluorescence tests with sera of mice bearing rat tumour xenografts against rat tumour cells*

| Tumour of serum-donor mice*: Type | No. cells injected | Age (days) | Mean diameter (cm) | FI *vs* cells of Sarcoma Mc7 | Hepatoma D23 |
|---|---|---|---|---|---|
| Sarcoma Mc7 | $10^6$ | 24 | 1·3 | 0·00 | 0·03 |
| | | 28 | 1·5 | 0·01 | 0·00 |
| | | 46 | 1·5 | 0·00 | 0·01 |
| | | 24 | 1·6 | 0·02 | 0·00 |
| | | 24 | 1·7 | 0·00 | 0·00 |
| Hepatoma D23 | $10^5$ | 27 | 1·2 | 0·01 | 0·02 |
| | | 32 | 1·5 | 0·00 | 0·00 |
| | | 35 | 1·0 | 0·01 | 0·01 |
| Heterozygous (*nu*/+) anti-rat | | | | 1·00, 1·00 | 0·98, 0·99 |

* Sera pooled from 1–4 mice.

TABLE III.—*Immunofluorescence tests with sera of athymic mice rejecting rat tumour cells injected in admixture with BCG*

| Inoculum rejected*: Tumour | No. cells | BCG (μg) | FI *vs* cells of Sarcoma Mc7 | Hepatoma D23 |
|---|---|---|---|---|
| Mc7 | $10^6$ | 500 | 0·00 | 0·02 |
| D23 | $10^5$ | 500 | 0·07 | 0·00 |
| D23 | †$10^5$ / $10^5$ | 500 / 500 | 0·00 | 0·01 |
| D23 | $10^5$ / $10^5$ | 500 / 500 | 0·00 | 0·01 |
| Heterozygous (*nu*/+) anti rat | | | 1·00 | 0·99 |

* Serum pooled from 1–3 mice.
† 2 injections at contralateral s.c. sites at 30-day intervals.

*Detection of tumour-specific antigen in the serum of xenograft-bearing mice*

The sera of athymic mice bearing xenografts were assayed for antigen by their ability to neutralize the reaction of rat tumour-immune antisera against appropriate target cells in the MIF test. The results of these assays (Table IV) showed

TABLE IV.—*Neutralization of rat tumour-immune serum by serum of athymic mice bearing tumour xenografts*

| Tumour of serum-donor mice* | | | | Immunofluorescence index of tumour-immune rat sera admixed with† | | |
|---|---|---|---|---|---|---|
| Type | No. cells injected | Age (days) | Mean diameter (cm) | — | Tumour-bearer mouse serum | Normal mouse serum |
| Mc7 | $10^6$ | 46 | 1·5 | 0·59 | 0·25 | — |
| | | | | 0·55 | 0·14 | |
| | | | | — | 0·18 | 0·59 |
| Mc7 | $10^6$ | 24 | 1·7 | 0·56 | 0·00 | 0·55 |
| Mc7 | $10^6$ | 28 | 1·4 | 0·44 | 0·01 | — |
| | | | | 0·65 | 0·11 | — |
| Mc7 | $10^6$ | 22 | 1·1 | 0·56 | 0·05 | — |
| | | | | 0·85 | 0·09 | — |
| | | | | 0·72 | 0·00 | 0·63 |
| Mc7 | $10^6$ | 23 | 1·3 | 0·59 | 0·45 | — |
| | | | | 0·59 | 0·47 | — |
| D23 | $10^5$ | 40 | 2·0 | 0·44 | 0·00 | — |
| D23 | $10^5$ | 26 | 1·2 | 0·67 | 0·00 | — |

* Sera pooled from 1–4 mice.

† 0·1 ml mouse serum added to 0·1 ml tumour-immune rat serum.

that 4/5 pools of sera from mice with Mc7 xenografts contained antigen, and both of 2 serum pools from D23 bearers were also positive. For example, in the first test, sera from mice with 46-day-old xenografts of Mc7 reduced the FI of Mc7 antiserum from 0·55–0·59 to 0·14–0·25, when 0·1 ml tumour-bearer serum was mixed with 0·1 ml antiserum, while normal mouse serum (*nu*/*nu*) had no neutralizing effect (FI of mixture, 0·59). The 2 D23 tumour-bearer sera reduced the FI of D23-immune serum from 0·44–0·67 to zero. A cross test carried out with the second of these D23 tumour-bearer sera showed that this neutralization

FIGURE.—Neutralization of Mc7 tumour-specific antiserum by sera of athymic mice bearing Mc7 xenografts. 0·1 ml aliquots of rat Mc7 tumour-specific antiserum were incubated with increasing volumes of serum from 3 mice bearing rat sarcoma Mc7 xenografts for 30 min at room temperature, and subsequently assayed for remaining free antibody by the indirect immunofluorescence test against sarcoma Mc7 cells.

of antiserum was specific, the D23-bearer serum failing to neutralize the reactivity of Mc7 antiserum against Mc7 target cells in 2 separate tests (FIs of untreated serum: 0·55, 0·72; FI after mixture with D23 bearer serum: 0·56, 0·63).

To determine the amount of tumour-bearer mouse serum required to neutralize a standard volume of tumour-immune serum, tests were carried out with 0·02–0·25 ml Mc7 xenograft bearer serum mixed with 0·1 ml rat tumour-immune serum (Fig.). With 3 individual tumour-bearer sera, increasing amounts of serum produced progressive neutralization of reactivity.

### DISCUSSION

Human tumours growing as xenografts in congenitally athymic or immunosuppressed mice have been reported to continue the production of characteristic tumour-associated secretory products. Thus, a xenograft of Burkitt's lymphoma continued the production of IgM (Povlsen *et al.*, 1973), human colon-tumour xenografts produce characteristic epithelial mucin (Houghton & Taylor, 1978*b*) CEA and AFP, these latter demonstrable both within the xenograft and free in the host serum (Mach *et al.*, 1974; Houghton & Taylor, 1978*b*; Miwa *et al.*, 1977). Similarly, a choriocarcinoma synthesized HCG (Kameya *et al.*, 1976), and human mammary carcinomas continue to produce calcitonin (Coombes *et al.*, 1975) and tumour-associated glycolipid (absent from non-malignant tissue) and shed this into the serum (Nordquist *et al.*, 1978). Similar studies with animal-tumour xenografts have demonstrated the synthesis of myeloma protein by a mouse plasmacytoma, insulin by a hamster pancreatic-islet-cell tumour, and SV40 T-antigen by SV40-transformed mouse, rat and rabbit cells (Freedman *et al.*, 1976). The objective of the present studies was to examine the continued production in xenografts of the unique cell-surface tumour-associated neoantigens of 2 carcinogen-induced rat tumours, and here it has been demonstrated that these antigens are expressed during xenograft growth. Moreover, free antigen, assayed by its ability to specifically neutralize tumour-specific antibody, was demonstrable in the serum of xenograft bearers. Antigen is similarly shed during growth of these tumours in syngeneic rats, but is then frequently complexed with tumour-specific antibody (Bowen & Baldwin, 1976; reviewed by Price & Baldwin, 1977). Assays carried out in the present study were with sera of mice with large tumours (1–2 cm in diameter) and no attempt has yet been made to correlate the level of serum tumour antigen and tumour size. It is probable that the serum of tumour-bearing mice contains other rat tumour products in addition to tumour-specific antigen, including species-specific antigens, but assays for these have not been carried out.

The present tests were unable to detect antibody to tumour-specific antigens or rat species antigens in the serum of mice developing xenografts. These findings do not exclude the possibility of the presence in tumour-bearer serum of antibody-antigen complexes in antigen excess, although mice rejecting 2 inocula of tumour cells prevented from growth by the local application of BCG, also had no detectable antibody. With virally induced tumours, athymic mice bearing growths of SV40-induced tumours similarly fail to develop antibody to SV40 T antigen, assayed by immunofluorescence reactions, or virus neutralizing antibody (Tevethia *et al.*, 1977) although inoculation of polyoma virus evokes antibody to virus, detectable by haemagglutination inhibition (Stutman, 1975). With human tumour xenografts, anti-human antibody was demonstrable in the serum of athymic mice with a Burkitt's lymphoma xenograft (Povlsen *et al.*, 1973) by both an indirect membrane-immunofluorescence test similar to that used here, and by complement-dependent cytotoxicity. However, antibody against membrane-associated EBV virus was not demonstrable, and the precise antigenic components of the human cells in the athymic mice were unresolved. Similar studies with mice bearing xenografts of the human bladder carcinoma line T24 failed to detect antibody by the membrane-immunofluorescence test (Pimm & Baldwin, 1978).

Essentially the present studies extend those of others who have demonstrated continued production or excretion of tumour-*associated* materials, showing that individually distinct tumour-*specific* components are also produced during xenograft growth. Taken as a whole, these studies indicate that many fundamental characteristics of malignant cells remain unchanged during xenograft development, strengthening the case that xenografts may be used as models for the assessment of therapeutic protocols and as a source of tumour tissue or antigen, and that the serum of tumour-bearing animals may be a suitable source of tumour products.

This work was supported by a grant from the Cancer Research Campaign, and was carried out with the skilled technical assistance of Mrs S. J. Wealthall and Mrs A. P. Hopper.

### REFERENCES

BALDWIN, R. W. & BARKER, C. R. (1967*a*) Tumour-specific antigenicity of aminoazo-dye-induced rat hepatomas. *Int. J. Cancer*, **2**, 355.

BALDWIN, R. W. & BARKER, C. R. (1967*b*) Demonstration of tumour-specific humoral antibody against aminoazo dye-induced rat hepatomata. *Br. J. Cancer*, **21**, 793.

BALDWIN, R. W. & PIMM, M. V. (1971) Influence of BCG infection on growth of 3-methylcholanthrene-induced rat sarcomas. *Eur. J. Clin. Biol. Res.*, **16**, 875.

BOWEN, J. G. & BALDWIN, R. W. (1976) Isolation and characterisation of tumour-specific antigen from the serum of rats bearing transplanted aminoazo dye-induced hepatomas. *Transplantation*, **21**, 213.

COOMBES, R. C., EASTY, G. C., DETRE, S. I. & 7 others. (1975) Secretion of immunoreactive calcitonin by human breast carcinomas. *Br. Med. J.*, iv, 187.

DAVY, M., BRUSTAO, T. & MOSSIGE, J. (1977) Irradiation of human ovarian tumors in nude mice. In *Proc. 2nd Int. Workshop on Nude Mice*. Eds T. Nomura, N. Ohsawa, N. Tamaoki & K. Fujiwara. Stuttgart: Guntar Fischer Verlag. p. 491.

FREEDMAN, V. H., BROWN, A. L., KLINGER, H. P. & SHIN, S. I. (1976) Mass production of animal cells in nude mice with retention of cell specific markers. *Exp. Cell Res.*, **98**, 143.

HOUGHTON, P. J., HOUGHTON, J. A. & TAYLOR, D. M. (1977) Effects of cytotoxic agents on TdR incorporation and growth delay in human colonic tumour xenografts. *Br. J. Cancer*, **36**, 206.

HOUGHTON, J. A. & TAYLOR, D. M. (1978*a*) Growth characteristics of human colorectal tumours during serial passage in immune-deprived mice. *Br. J. Cancer*, **37**, 213.

HOUGHTON, J. A. & TAYLOR, D. M. (1978*b*) Maintenance of biological and biochemical characteristics of human colorectal tumours during serial passage in immune-deprived mice. *Br. J. Cancer*, **37**, 199.

KAMEYA, T., SHIMOSATO, Y., TUMURAYA, M., OHSAWA, N. & NOMURA, T. (1976) Human gastric choriocarcinoma serially transplanted in nude mice. *J. Natl Cancer Inst.*, **56**, 325.

KOPPER, L. & STEEL, G. G. (1975) The therapeutic response of three human tumor lines maintained in immune-suppressed mice. *Cancer Res.*, **35**, 2704.

LAMERTON, L. F. & STEEL, G. G. (1975) Growth kinetics of human large bowel cancer growing in immune-depressed mice and some chemo-therapeutic observations. *Cancer*, **36**, 2431.

MACH, J. P., CARREL, S., MERENDA, C., SORDAT, B. CEROTTINI, J. C. (1974) *In vivo* localisation of radiolabelled antibodies to carcinoembryonic antigen in human colon carcinoma grafted into nude mice. *Nature*, **248**, 704.

MIWA, M., HIROHASHI, S., SHIMOSATO, Y. & 5 others. (1977) Coproduction of carcinoembryonic antigen and $\alpha$-fetoprotein in transplantable human colon cancer in nude mice. In *Proc. 2nd International Workshop on Nude Mice*. Eds T. Nomura, N. Ohsawa, N. Tamaoki, K. Fujiwara. Stuttgart: Gunter Fischer Verlag. p. 435.

NORDQUIST, R. E., ANGLIN, J. M. & LERNER, M. P. (1978) Antigen shedding by human breast-cancer cells *in vitro* and *in vivo*. *Br. J. Cancer*, **37**, 776.

PICKARD, R. G., COBB, L. M. & STEEL, G. G. (1975) The growth kinetics of xenografts of human colorectal tumours in immune deprived mice. *Br. J. Cancer*, **31**, 36.

PIMM, M. V. & BALDWIN, R. W. (1975) BCG Immunotherapy of rat tumours in athymic nude mice. *Nature*, **254**, 77.

PIMM, M. V. & BALDWIN, R. W. (1978) BCG Treatment of human tumour xenografts in athymic nude mice. *Br. J. Cancer*, **38**, 699.

POVLSEN, C. O., FIALKOW, P. J., KLEIN, E., KLEIN, G., RYGAARD, J. & WIENER, F. (1973) Growth and antigenic properties of a biopsy-derived Burkitt's lymphoma in thymic-less (nude) mice. *Int. J. Cancer*, **11**, 30.

POVLSEN, C. O. & JACOBSEN, G. K. (1975) Chemotherapy of a human malignant melonoma transplanted in the nude mouse. *Cancer Res.*, **35**, 2790.

PRICE, M. R. & BALDWIN, R. W. (1977) Shedding of tumour cell surface antigens. In *Dynamic Aspects of Cell Surface Organisation. Cell Surface Reviews*. Vol. 3. Eds G. Porte & G. L. Nicholson. Amsterdam: Elsevier. p. 423.

REEVES, B. R. & HOUGHTON, J. A. (1978) Serial cytogenetic studies of human colonic tumour xenografts. *Br. J. Cancer*, **37**, 612.

SONIS, S. T., FALCAO, R. P. & MACLENNAN, I. C. M. (1977) Assessment of drug sensitivity of human leukaemic myeloblasts. II. The toxic effects of cytosine arabinoside on $^{125}$IUdR-labelled human leukaemic myeloblasts in mice. *Br. J. Cancer*, **36**, 307.

STUTMAN, O. (1975) Tumor development after polyoma infection in athymic nude mice. *J. Immunol.*, **114**, 1213.

TEVETHIA, S. S., WANECK, G. & TEVETHIA, M. (1977) Immune response of athymic nude mice to papovavirus SV40 tumour associated antigens. *Int. J. Cancer*, **19**, 700.